# Analysis of Mammalian Cell Proliferation and Macromolecule Synthesis Using Deuterated Water and Gas Chromatography-Mass Spectrometry

**DOI:** 10.3390/metabo6040034

**Published:** 2016-10-13

**Authors:** Victoria C. Foletta, Michelle Palmieri, Joachim Kloehn, Shaun Mason, Stephen F. Previs, Malcolm J. McConville, Oliver M. Sieber, Clinton R. Bruce, Greg M. Kowalski

**Affiliations:** 1 Institute for Physical Activity and Nutrition Research, School of Exercise and Nutrition Sciences, Deakin University, Geelong 3216, Victoria, Australia; victoria.foletta@deakin.edu.au (V.C.F.); s.mason@deakin.edu.au (S.M.); clinton.bruce@deakin.edu.au (C.R.B.); 2 Systems Biology and Personalised Medicine Division, The Walter and Eliza Hall Institute of Medical Research, Parkville 3052, Victoria, Australia; palmieri.m@wehi.edu.au (M.P.); sieber.o@wehi.edu.au (O.M.S.); 3 Department of Medical Biology, University of Melbourne, Parkville 3052, Victoria, Australia; 4 Department of Biochemistry and Molecular Biology, Bio21 Molecular Science and Biotechnology Institute, University of Melbourne, Parkville 3052, Victoria, Australia; j.kloehn@student.unimelb.edu.au (J.K.); malcolmm@unimelb.edu.au (M.J.M.); 5 MRL, Merck & Co. Inc., Kenilworth, NJ 07033, USA; stephen_previs@merck.com; 6 Department of Surgery, University of Melbourne, Parkville 3052, Victoria, Australia; 7 School of Biomedical Sciences, Monash University, Clayton 3168, Victoria, Australia

**Keywords:** deuterated water, biomass, GC-MS, stable isotopes, C2C12, colon cancer, protein synthesis, lipogenesis, DNA synthesis

## Abstract

Deuterated water (^2^H_2_O), a stable isotopic tracer, provides a convenient and reliable way to label multiple cellular biomass components (macromolecules), thus permitting the calculation of their synthesis rates. Here, we have combined ^2^H_2_O labelling, GC-MS analysis and a novel cell fractionation method to extract multiple biomass components (DNA, protein and lipids) from the one biological sample, thus permitting the simultaneous measurement of DNA (cell proliferation), protein and lipid synthesis rates. We have used this approach to characterize the turnover rates and metabolism of a panel of mammalian cells in vitro (muscle C2C12 and colon cancer cell lines). Our data show that in actively-proliferating cells, biomass synthesis rates are strongly linked to the rate of cell division. Furthermore, in both proliferating and non-proliferating cells, it is the lipid pool that undergoes the most rapid turnover when compared to DNA and protein. Finally, our data in human colon cancer cell lines reveal a marked heterogeneity in the reliance on the de novo lipogenic pathway, with the cells being dependent on both ‘self-made’ and exogenously-derived fatty acid.

## 1. Introduction

The use of radioactive and stable isotope tracers to measure metabolic processes has underpinned many fundamental discoveries in biochemistry and physiology [1]. Unlike ‘static’ measurements of metabolite concentration, the use of tracers adds the dimension of time, allowing inferences to be made about reaction rates and metabolic fluxes [1,2,3]. This kinetic feature permits the observation and quantification of biochemical networks and biological processes, thus providing a detailed functional readout of the behavior of the biological system [2,3]. In essence, metabolic pathway fluxes represent the functional output of multiple nonlinear regulatory layers that include gene, protein and metabolite interactions [4].

To gain the most insight out of any experiment, it is ideal to be able to probe multiple pathways simultaneously, thus allowing a broad assessment of cellular behavior. To do this, one would ideally use a near-universal tracer that simultaneously labels multiple cellular metabolites (which can be detected as free metabolites or constituents of macromolecules). In this regard, deuterated ‘heavy’ water (^2^H_2_O) is a particularly useful metabolic tracer [2,3]. Over 70 years ago, it was discovered that introduction of low concentrations of ^2^H_2_O into biological systems results in the incorporation of deuterium (^2^H) into a wide range of cellular metabolites and corresponding macromolecules containing these metabolites [5,6,7,8,9,10,11,12,13,14]. Following the administration of ^2^H_2_O into the body water pool in vivo or into cell culture media in vitro, ^2^H atoms become stably incorporated via enzyme catalyzed reactions into C-H bonds of nonessential amino acids, glycerol-3-phosphate, fatty acids, cholesterol, hexoses and pentoses (ribose and deoxyribose). Subsequent analysis of ^2^H labelling of these constituents in macromolecules provides a highly sensitive measure of the rates of synthesis/turnover of lipids [15,16,17,18,19,20,21,22,23,24,25,26,27], proteins [15,28,29,30,31,32,33,34], DNA [15,35,36], RNA [15] and glucose/glycogen [25,26,37,38,39,40,41,42,43] and an objective assessment of fundamental cellular processes that include, but are not limited to, rates of cell and organelle proliferation, cell and tissue biomass turnover, de novo lipogenesis and gluconeogenesis. Such measurements have tremendous application to researchers in the field of cancer, immunology, cardiovascular disease, metabolism, diabetes, obesity, developmental biology, as well as the biotechnology and agricultural sectors. In addition to measuring multiple metabolic processes simultaneously, ^2^H_2_O labelling is advantageous because: (1) the even distribution of the ^2^H_2_O in the total body/cellular pool makes it simple to determine the precursor:product labelling ratios that are required to calculate synthetic flux with confidence; (2) the ^2^H_2_O tracer can be easily and safely administered for long periods of time in vivo and in vitro, permitting flux measurements in free living conditions or long-term culture experiments; and (3) the capacity to label for extended periods of time leads to efficient labelling of total cellular biomass, increasing the sensitivity of the analysis and reducing artefacts that are sometimes associated with incomplete quenching of cells/tissues during harvesting [3,24,32,35].

In most published studies, ^2^H_2_O labelling protocols generally focus on measuring ^2^H incorporation into a single class of macromolecules, and/or replicate samples are prepared for parallel extractions of different classes of macromolecules. However, the latter approach is not always possible if sample is limiting. We therefore present here a simple method that simultaneously extracts nucleic acid, protein, lipid and polar metabolite fractions from a single sample, enabling simultaneous measurement of synthetic rates of different biomass components using ^2^H_2_O and gas chromatography coupled to mass spectrometry (GC-MS). We have used this method to characterize cellular biomass synthetic fluxes in a panel of mammalian cells in vitro, thus demonstrating the utility of this approach.

## 2. Methods

**Figure 1:**
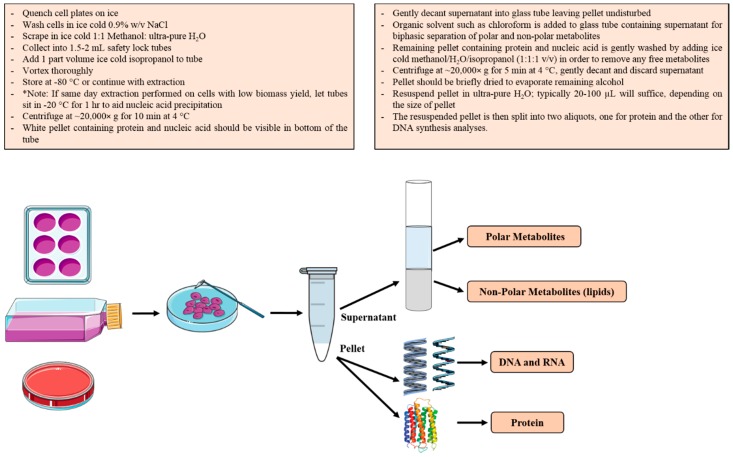
Schematic representation and general steps for the single sample biomass fractionation/extraction method.

We have developed a method for sequentially extracting the major classes of macromolecules from mammalian cells, although this method can be readily adapted to other eukaryotic cells that lack a cell wall (Figure 1). We have found this method to be easily scalable depending on sample availability and instrument sensitivity. The method is based on that described by Sapcariu et al. [44], but generates a pellet containing protein and DNA/RNA after the initial organic solvent extraction, rather than an interphase precipitate [44], facilitating the separation of protein/nucleic acid from polar and non-polar metabolites. Furthermore, the method exploits a simple cell quenching protocol involving rapid chilling of cultures/tissues on ice, which is sufficient to prevent the turnover of macromolecules of interest during the initial extraction.

### 2.1. Cell Culture

The mouse C2C12 myoblast cell line was obtained from ATCC (Manassass, VA, USA). Human cancer cell lines (colon carcinoma: DLD1, SW480, SKCO1 and LOVO) were obtained from ATCC and genetically validated at the Australian Genome Research Facility at the Walter and Eliza Hall Institute of Medical Research. Dulbecco’s Modified Eagle’s Medium (DMEM), fetal bovine serum (FBS), horse serum, Dulbecco’s phosphate-buffered saline without calcium or magnesium (DPBS) and cell trypsinizing agent TrypLE™ were from Life Technologies (ThermoFisher Scientific, Bayswater, VIC, Australia). Corning tissue culture plastic-ware was purchased from In Vitro Technologies (Noble Park, VIC, Australia). Deuterated water (deuterium oxide; ^2^H_2_O) was obtained from Sigma-Aldrich (St. Louis, MO, USA). C2C12 myoblasts and colon carcinoma lines were cultured in 25 mM d-glucose (high glucose, 4.5 g/L) DMEM containing glutamine, sodium pyruvate and HEPES (Life Technologies; 11995-073) supplemented with 10% v/v FBS (growth medium). All experiments were performed on myoblasts cultured between Passages 4–10. To differentiate the myoblasts, cells were grown to confluence, and the medium was changed to high glucose DMEM supplemented with 2% v/v horse serum (differentiation medium). Growth and differentiation media were replenished every 48 h.

### 2.2. ^2^H_2_O Labelling

Experiment 1: C2C12 myoblasts were initially plated at 2 × 10^4^ cells/mL in a 6-well plate in duplicate before being suspended in growth medium containing 4% ^2^H_2_O and harvested at 12, 24, 48, 72, 96 and 120 h (with fresh ^2^H_2_O media replacement at the 48- and 96-h time-points). Note: after reaching the 72 h time-point, the cells destined for the 96 and 120 h treatment were subcultured (replated) at a lower density in the 4% ^2^H_2_O containing media in order to remain subconfluent and hence in a proliferative state. Duplicate wells containing no cells were also filled with 4% ^2^H_2_O labelled media and harvested at 96 h to provide extraction blank samples in order to determine if any contamination was present (particularly with lipids). A parallel plate was also prepared in which cells were incubated in standard growth media (non-^2^H_2_O) allowing for correction of natural isotopic background abundance in the different macromolecular constituents. Experiment 1 was used to initially establish the time course required to achieve plateau ^2^H enrichments in DNA and protein in the C2C12 myoblasts, thus permitting the calculation of proliferation and protein turnover rates.

Experiment 2: An independent ^2^H_2_O labelling experiment was undertaken to assess the reproducibility of the method and to detect fundamentally different biological behaviors in the same cell type. Specifically, actively dividing C2C12 cells (proliferating myoblasts) and post-mitotic C2C12 cells that had been terminally differentiated into myotubes were labelled as described in Experiment 1 (although only including 12-, 24-, 48- and 96-h time-points). For labelling of the C2C12 myotubes, myoblasts were plated as before; however, upon reaching confluence, myoblast cells were allowed to differentiate for two days prior to the addition of 4% ^2^H_2_O differentiation medium to commence the 12–96-h labelling experiment. Macromolecular turnover was assessed by measuring ^2^H enrichment in DNA-derived deoxyribose (proxy for cell proliferation rate), protein-derived alanine (proxy for protein synthesis/turnover rates), total cellular palmitate (proxy for de novo lipogenesis) and lipid-derived glycerol in the total lipid pool (proxy for membrane turnover).

Experiment 3: Triplicate wells were plated with DLD1, SW480, SKCO1 and LOVO cells (1 × 10^4^ cells/well) in DMEM with 10% FBS. Seventy-two hours post plating, ^2^H_2_O media (DMEM with 5% ^2^H_2_O) were added over a time course of 8, 24, 48, 72 and 96 h with the same extraction blank and ‘no labelling’ controls as described in Experiment 1.

### 2.3. Biomass Fractionation

The general workflow for this procedure is described and schematically depicted in Figure 1. All solvents were analytical grade, and ultra-pure H_2_O was used. Culture plates were placed on ice-water slurry and the media rapidly aspirated, prior to washing of the cell monolayers with ice cold 0.154 M NaCl (0.9% w/v). Cells were subsequently covered in ice-cold methanol/H_2_O (1:1 v/v, 800 μL) then scraped and transferred to Eppendorf tubes. Protein and nucleic acids in the suspension were precipitated by the addition of ice-cold isopropanol (400 μL) to generate a single phase solution (methanol/H_2_O/isopropanol; ~1:1:1 v/v). At this stage, the samples were frozen at −80 °C (optional). The suspension, containing flocculent material, was centrifuged for 10 min at 20,000× g and the clear supernatant (containing polar and non-polar metabolites) transferred to a clean glass screw cap tube (100 × 13 mm Kimax^®^ Test Tube). The single phase supernatant was converted to a two-phase system by the addition of 400 μL chloroform. Following gentle centrifugation, the upper phase containing polar metabolites was removed and discarded, while the bottom organic layer (isopropanol and chloroform) was transferred to another clean glass tube and evaporated to complete dryness in a speed vacuum (Labconco, Kansas, MO, USA) for analysis of total lipids. The presence of polar metabolites in the upper phase of the cell supernatant was confirmed via GC-MS using previously published methods [45,46] (Figure S1).

The first pellet fraction generated in the Eppendorf tube was gently washed by adding ice cold methanol/H_2_O/isopropanol (1:1:1 v/v 600 μL) and centrifuged for 5 min at 20,000× g. The supernatant was removed by gentle decanting and the pellet dried by leaving the tubes open in a fume cupboard for 15–30 min; to ensure the supernatant is removed gently, as the pellet can be unstable. After drying, the pellet was resuspended in ultra-pure H_2_O (50 μL), heated at 60 °C for 10 min followed by thorough vortexing. Note: heating is required to resuspend the pellet, and this can be further enhanced by immediate vortexing and or pipette mixing. The larger the pellet, the greater the volume of H_2_O that could be required for resuspension (i.e., add extra H_2_O in 25 μL increments till the pellet is resuspended). An aliquot (20 μL) of the suspension was transferred to a clean safety lock Eppendorf tube (for measurement of protein synthesis), while the remaining 30 μL of the mixture was used for the analysis of DNA synthesis.

### 2.4. Analysis of Deuterium Incorporation into Protein

The protein sample (20 μL) was diluted with 400 μL of 6 M HCl and hydrolyzed by incubation at 110 °C overnight. Following the acid hydrolysis, the samples were evaporated to complete dryness in an acid-resistant speed vacuum (Labconco, Kansas, MO, USA) at 60 °C. The released amino acids were derivatized by the addition of pyridine (50 μL, Sigma) and MTBSTFA + 1% TBDMCS (50 μL, Sigma), and incubated at 60 °C for 30 min. The mixture was transferred into 250-μL glass inserts (Agilent) in 2-mL glass vials and analyzed by GC-MS. The ^2^H incorporation into protein-derived alanine was used to determine the protein synthesis rates.

### 2.5. Analysis of Deuterium Incorporation into DNA

The nucleic acid sample (30 μL) was suspended in 250 μL proteinase K solution (0.2 mg/mL) and incubated at 55 °C overnight. The proteinase K solution was made from a 1-mg/mL H_2_O stock of proteinase K (Sigma) mixed with VIAGEN Direct PCR Tail Lysis Reagent (1:4 v/v proteinase K: lysis buffer). The protein-free solution was diluted with isopropanol (250 μL), and samples were centrifuged at 20,000× g for 5 min at room temperature, to precipitate DNA. The supernatant was carefully discarded, the DNA pellet washed with 70% ethanol (500 μL, 2 min) and the supernatant removed after centrifugation at 20,000× g for 5 min. The DNA pellet was left to dry for ~15 min, then resuspended in H_2_O (200 μL) with heating at 55 °C for 10 min followed by vortexing. The resuspended DNA samples were hydrolysed and dephosphorylated by the addition of 50 μL of 5× hydrolysis buffer containing S1 nuclease (Sigma; N5661; 50 kU) and potato acid phosphatase (Merck Millipore/Calbiochem; 524529; 1 kU) and incubated overnight at 37 °C [15,35]. The complete (enzyme containing) 5× hydrolysis buffer was prepared by initially making a 100-mL 5× buffer stock containing 0.375 M of sodium acetate (pH 5.0; adjusted with glacial acetic acid) and 1 M ZnSO_4_.7H_2_O. The potato acid phosphatase was then resuspended in 1 mL of ultra-pure H_2_O while the S1 nuclease in 2 mL of 1× hydrolysis buffer (diluted ZnSO_4_ containing 5× hydrolysis buffer). Following reconstitution, both enzymes were completely transferred from their original containers into 37 mL of 5× ZnSO_4_ containing hydrolysis buffer, making a final volume of ~40 mL. This complete 5× hydrolysis buffer was aliquoted into Eppendorf tubes (1 mL each) and stored at −20 °C for subsequent use, with each 1-mL aliquot providing ~20 reactions. After DNA hydrolysis, the deoxyribose moiety was derivatized in a two-step reaction. First, 0.184 M HCl (100 μL) was added to the 250 μL hydrolysed sample followed by the addition of 10 μL of 50 mM *O*-(2,3,4,5,6-pentafluorobenzyl) hydroxylamine hydrochloride (PFHBA, 12.5 mg/mL H_2_O; Sigma) and the reaction incubated at 90 °C for 3 h. The oxime of deoxyribose was extracted by the addition of ~20 mg of NaCl (aids biphasic partitioning) and 1 mL of ethyl acetate/hexane mix (1:1 v/v) to each tube. The samples were vigorously vortexed and centrifuged at 2500× g for 2 min. A portion (600 μL) of the top organic phase was carefully transferred to a new 12 × 75-mm glass Kimble tube. A second extraction was then performed on the original sample by the addition of 800 μL of pure ethyl acetate followed by vortexing and centrifugation, as in the previous step. In the second extraction 750 μL of the top organic phase was carefully transferred to the previously extracted sample in the corresponding Kimble tube. The pooled samples were then dried in a speed vacuum at 30 °C. Once dry, samples were resuspended in 50 μL of pyridine and 50 μL acetic anhydride (Sigma), thoroughly vortexed and the mixture transferred into 2-mL GC vials with 250-μL inserts and heated at 60 °C for 30 min. This derivatization method forms the perfluorotriacetyl derivative of deoxyribose and permits analysis via GC-MS in the negative chemical ionization (NCI) mode [35]. Analysis in the NCI mode increases analytical sensitivity and allows DNA synthesis rates to be performed in the presence of relatively low yields of DNA [35]. The deuterium incorporation into DNA-derived deoxyribose was used to quantify DNA synthesis rates and hence cell doubling times [35].

### 2.6. Analysis of Deuterium Incorporation into Lipids

The dried organic phase (total cellular lipids) was transesterified by the addition of 3N methanolic HCl (250 μL, Sigma) and incubation at 60 °C for 1 h. This reaction releases all esterified fatty acids, with the formation of the corresponding fatty acid methyl esters (FAME) that are amenable to analysis by GC-MS. The methanolic HCl was evaporated to dryness under a gentle stream of nitrogen gas and samples resuspended in pyridine (50 μL) and acetic anhydride (50 μL), vortex mixed and transferred into 250-μL glass inserts in 2-mL GC vials and heated at 60 °C for 30 min. This second reaction was used to convert lipid-derived glycerol molecules obtained from the transesterification process into the glycerol triacetate derivative. Total lipid synthesis/turnover rates and de novo lipogenesis (DNL) were measured by measuring the incorporation of ^2^H into lipid-derived glycerol and palmitate (C16:0), respectively. Palmitate labelling is used for DNL measurements, as it is the major product of the fatty acid synthase reaction in mammals, with labelling in fatty acids that are >16 carbons in length resulting from chain elongation, not DNL [22,27]. The major advantage of using ^2^H_2_O to measure DNL is that it accounts for all substrate sources used to make fatty acids (all source DNL), as opposed to providing a readout of the contribution of only one specific substrate (precursor) to the newly-synthesized fatty acid, as is the case with other tracers, such as glucose and glutamine, etc. Note: palmitate contamination is a common occurrence, particularly if plastic consumables are used with solvents. One should ensure glassware is used where possible and multiple extraction blanks incorporated into each experiment. The use of multiple extraction blanks can determine the degree of palmitate contamination. A high palmitate background will lead to an underestimation of palmitate enrichment in biological samples and may introduce a high variability in labelling between biological replicates.

### 2.7. GC-MS

Total protein synthesis/turnover data were generated using an Agilent 6890N GC system and an Agilent 5975C MSD (Agilent Technologies, Santa Clara, CA, USA) in electron ionization (EI) mode, with helium as the carrier gas. Specifically the 260 *mz* (M_0_), 261 *mz* (M_1_) and 262 *mz* (M_2_) ions of the alanine *tert*-butyldimethylsilyl (t-BDMS) derivative were analyzed in selective ion monitoring mode (SIM). These specific ions retain the entire molecular structure of the alanine molecule, permitting every possible deuterium enrichment in the available alanine C-H bonds to be measured. A VF-5 capillary column with a 10-m inert EZ-guard (J & W Scientific, 30 m, 0.25 mm, 0.25 µm) was used, and the front inlet and transfer line temperatures were both set to 270 °C, while the quadrupole and source temperatures were set to 150 °C and 230 °C, respectively. The oven temperature gradient was set to: 100 °C (2 min); 100 °C–320 °C at 25 °C/min with a 3-min hold time at 320 °C. The sample (1 μL) was injected with a 20:1 split ratio. Note: for GC-MS tracer isotopic enrichment analysis, the split ratio setting should be modified accordingly to the intensity of the chromatographic peak, ensuring that the GC column or MSD are not overloaded (within linear dynamic range) and that the chromatogram shape is sharp, symmetrical and front or back end tailing not present, as a high quality chromatographic separation and peak shape are required for accurate tracer enrichment analysis. Performing an analysis with unlabeled chemical standards over a range of concentrations can easily determine the dynamic linear range for the specific molecule of interest.

DNA synthesis data were generated using an Agilent 7890B GC system and an Agilent 5977B MSD in NCI mode, with helium as the carrier and methane as the reagent gas. The perfluorotriacetyl derivative of deoxyribose was analyzed by monitoring the 435 *mz* (M_0_), 436 *mz* (M_1_) and 437 *mz* (M_2_) ions in the SIM mode. Note: this derivative results in the formation of two deoxyribose peaks (*cis*-, and *trans*- isomers); typically the larger later eluting peak is used for quantification, although if chromatogram peaks are of high quality, both should yield near identical enrichments [35]. These specific ions retain all available deoxyribose C-H bonds [35]. A VF-5 capillary column with a 10-m inert EZ-guard (J&W Scientific, 30 m, 0.25 mm, 0.25 µm) was used, and the front inlet and transfer line temperatures were set to 280 °C and 250 °C, respectively, while the quadrupole and source temperatures were both set to 150 °C. The oven temperature gradient was set to: 50 °C (2.25 min); 50 °C–320 °C at 40 °C/min with a 3-min hold time at 320 °C. The sample (1 μL) was injected with a 10:1 split ratio. Note: As discussed above for alanine analysis, the split ratio setting should be optimized for the peak shape and avoidance of overloaded chromatograms. Analysis in the NCI mode is particularly sensitive, thus high DNA yields will require greater split ratio settings and or sample dilution.

Lipid synthesis data were generated using the same instrument and column as for DNA synthesis; however, the analysis was performed in positive chemical ionization (PCI) mode. Glycerol triacetate was analyzed by monitoring the 159 *mz* (M_0_), 160 *mz* (M_1_) and 161 *mz* (M_2_) ions and methyl palmitate using the 299 *mz* (M_0_), 300 *mz* (M_1_) and 301 *mz* (M_2_) ions. Both glycerol triacetate and methyl palmitate were analyzed within the one single run in the SIM mode. These specific ions retain all available C-H bonds within the glycerol and palmitate molecules. The front inlet and transfer line temperatures were set to 275 °C and 250 °C, respectively, while the quadrupole and source temperatures were set to 150 °C and 300 °C, respectively. The oven temperature gradient was set to: 60 °C (1.5 min); 60 °C–320 °C at 35 °C/min with a 3-min hold time at 320 °C. The sample (1 μL) was injected with a 10:1 split ratio. Again, as discussed earlier, the split ratio should be optimized for each experiment.

### 2.8. Calculations

The abundance of each chromatographic peak was calculated by integrating the area under the curve (AUC) for each specific ion using Agilent Mass Hunter Quantitative analysis software. To calculate sample enrichment, the natural isotopic background abundance of each sample needs to be subtracted. Therefore, sample enrichment in excess of background enrichment was calculated by applying the following equation to the calculated AUC values:

EM_1_ [Excess molar enrichment (%)] = [M_1_/(M_0_ + M_1_)_(biologicalsample)_ − M_1_/(M_0_ + M_1_)_(unlabeled sample)_] × 100 (1)

The cells not treated with ^2^H_2_O served as the reference unlabeled background control samples. To ensure there was no unexpected ion contamination in the biological samples (matrix effect), an unlabeled set of chemical standards for each metabolite was measured alongside each run. The natural isotopic background abundance of each chemical standard was equal to that of the corresponding unlabeled biological background control, such that:

[M_1_/(M_0_ + M_1_)]_(unlabeled sample)_ = [M_1_/(M_0_ + M_1_)]_(chemical standard)_ (2)

On the occasion that the percentage of turnover was shown, the calculation was made as follows:

Turnover (%) = EM_1(sample)_/EM_1(max)_ × 100 (3)

where EM_1(max)_ represents the excess M_1_ isotopomer enrichment in the fully-labelled (turned over) metabolite pool, also known as the asymptotic or maximal/plateau value.

To determine the fractional synthesis rate constant (*k*) and half-life (*t*_1/2_) of each specific biomass pool, rise-to-plateau kinetics were used, whereby non-linear (exponential precursor-product equation) least squares fitting plots were applied to the enrichment data over time:

F_(*t*)_= f_(max)_ × (1 − e*^−kt^*); or this can be expressed as EM_1(*t*)_ = EM_1(max)_ × (1 − e*^−kt^*) (4)

where F_(*t*)_ or EM_1(*t)*_ represent the fractional or percent ^2^H enrichment in a specific metabolite (i.e., alanine, glycerol, deoxyribose or palmitate) at a specific time during the labelling period (*t*), f_(max)_ or EM_1(max)_ represents the asymptotic (maximal or plateau) value in fractional or percentage units and *k* is the mathematically-predicted fractional synthesis rate constant (expressed in units of time^−1^). In these specific experiments, the culture times were measured in hours; thus, *k* was expressed as h^−1^.

Half-life (*t*_1/2_) represents the time taken for the specific metabolite pool to be half turned over, i.e., the time taken to achieve 50% of EM_1(max)_. In the case of DNA synthesis, the *t*_1/2_ of enrichment in DNA-derived deoxyribose represents the cell doubling time, i.e., after one cell division event, two DNA strands have been duplicated, resulting in two original DNA strands and two new ones (50% unlabelled and 50% labelled) [35]. Thus, the lower the half-life, the more rapid the rate of synthesis is. The half-life is calculated by the following equation:

Half-life (t_1/2_) = 0.693/*k* (5)

To determine what proportion or percentage of the total esterified palmitate pool was derived from DNL, it is necessary to perform mass isotopomer distribution analysis (MIDA). MIDA, is a combinatorial method (binomial distribution model) used to determine the maximum number of exchangeable carbon bound hydrogens (*N*) in a specific molecule [16,17,18,22,23,47]. While beyond a detailed discussion here, the requirement to perform MIDA on the palmitate data (and many other lipid classes also) results from the fact that in the presence of ^2^H_2_O, the total intracellular palmitate pool is a combination (mixture) of both labelled ‘self-made’ palmitate molecules and unlabeled pre-existing (exogenous) ones derived from the serum in the cell culture media. Thus, unlike in the other biomass pools analyzed here (i.e., protein-derived alanine, DNA-derived deoxyribose, etc.), the experimentally-obtained maximal (plateau) ^2^H enrichment values in palmitate do not necessarily correspond to the theoretical EM_1(max)_ [16,17,18,22,23,48]. The experimentally-determined plateau enrichment values indicate that the DNL-derived palmitate pool is fully turned over (replaced). However, the specific enrichments have not reached their absolute theoretical maximum as the uptake and esterification of unlabeled media-derived exogenous palmitate continually dilute the labelled ‘self-made’ pool. To determine the *N* value and hence true theoretical EM_1(max)_ for labelled palmitate from the experimental data, one can use the ratio of consecutive isotopomers (M_2_/M_1_) and the enrichment of ^2^H_2_O in the culture media (or body water pool in vivo). However, prior to calculating *N*, the raw M_1_ and M_2_ palmitate isotopomer values obtained from the GC-MS must be corrected for natural isotopic background abundance skew. Corrections were made using the matrix method described by Lee et al. [23]. The corrected data are expressed in the mole percent excess (MPE) form, whereby M_0_ + M_1_ + M_2_ = 100% or 1 if expressed in the fractional form. *N* was calculated using the following equation:

M_2_/M_1_ = [(*N*-1)/2]/[media ^2^H_2_O enrichment/(1 − media ^2^H_2_O enrichment] (6)

Once *N* was calculated for each experimental time point, the maximal theoretical enrichment, EM_1(max)_ was calculated as follows:

Theoretical EM_1(max)_ = *N* × media ^2^H_2_O enrichment (7)

Culture media ^2^H_2_O enrichment in C2C12 experiments was 4% (0.04), while in colon cancer cell experiments, it was 5% (0.05).

Finally, with the theoretical EM_1(max)_ solved, the percentage of newly-synthesized palmitate was then calculated by comparing the experimentally-observed total enrichment at each specific time point MPE_total(*t*)_ to the theoretical EM_1(max)_, such that:

Newly synthesized palmitate (%) = MPE_total(*t*)_/theoretical EM_1(max)_ × 100 (8)

MPE_total(*t*)_ =M_1_ × 1 + M_2_ × 2 (9)

Given the low amount of ^2^H_2_O (precursor) used in this study, M_3_ enrichments were negligible, and therefore, only M_1_ and M_2_ enrichments were used in the calculation of MPE_total(*t*)_. The MPE_total(*t*)_ is analogous to a specific activity in radiolabeled tracer terms and represents the observed total number of ^2^H atoms incorporated per palmitate molecule at any given experimental time.

## 3. Results and Discussion

### 3.1. Establishing a DNA and Protein ^2^H Labelling Time Course in C2C12 Myoblasts

**Figure 2:**
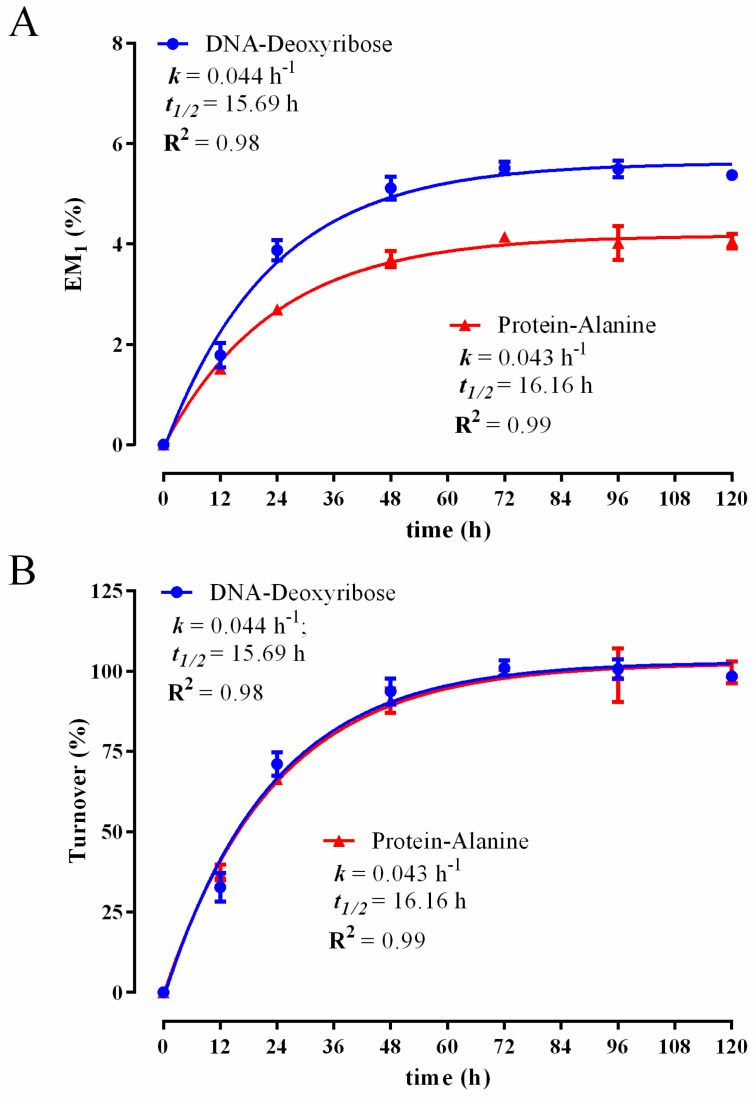
Time course for ^2^H-incorporation into DNA-derived deoxyribose and protein-derived alanine in C2C12 myoblasts following incubation in 4% ^2^H_2_O over 120 h ^2^H_2_O treatment. Excess molar enrichment in the M_1_ isotopomer (EM_1_) over time in DNA-derived deoxyribose and protein-derived alanine (**A**); the percent turnover of the cellular DNA and protein pools over time (**B**). Inset: fractional synthesis rate constant (*k*), half-life (*t*_1/2_) and goodness-of-fit (R^2^) from non-linear curve fitting. Two replicates were performed for each time point. Error bars represent the standard error of the mean (SEM).

Experiment 1 validated the biomass fractionation procedure (Figure 1) and the use of ^2^H_2_O labelling combined with the rise to plateau kinetics to simultaneously determine DNA and protein synthesis rates in rapidly-dividing cells in vitro. The murine C2C12 myoblast line rapidly proliferates under serum stimulated conditions, but can also be induced to undergo terminal differentiation. Differentiation is induced by replacing 10% FBS with 2% horse serum (mild serum starvation) when myoblasts reach confluence, which causes rapidly dividing C2C12 myoblasts to exit the cell cycle and fuse together to form multinucleated myotubes, a form of immature muscle fiber [48]. To initially establish the speed at which DNA and protein turnover were occurring under rapidly dividing conditions (10% FBS), we performed a broad labelling time course whereby subconfluent C2C12 myoblasts were treated with media containing 10% FBS in the presence of 4% ^2^H_2_O for a 12–120-h period. As seen in Figure 2A, ^2^H enrichment in DNA-derived deoxyribose and protein-derived alanine occurred rapidly, and while the absolute amount of labelling differed between the two molecules, the rate of rise in enrichment, referred to as the rise to plateau, was remarkably similar and occurred within 72 h. Accordingly, using non-linear least squares fitting of plots, the calculated DNA (*k* = 0.044 h^−1^) and protein (0.043 h^−1^) turnover constants were very similar. The *t*_1/2_ for protein, which represents the time taken to achieve 50% turnover, was 16.16 h. In the case of DNA labelling, the *t*_1/2_, which represents the cell doubling time, was calculated to be 15.69 h, which is consistent with previously published C2C12 doubling times of 15.2–19.7 h [49], as assessed using manual cell counting. The maximum level of ^2^H-labeling in deoxyribose was higher than in alanine (Figure 2A), because there are more opportunities for ^2^H to be incorporated into deoxyribose (through the pentose phosphate pathway, hexose phosphate isomerization) [35], compared to alanine (transamination of pyruvate) [30,31,32]. The synthesis rate is therefore determined by empirically measuring the time taken to achieve the unique maximal (plateau) labelling value, known as the rise to plateau. The enrichment data in Figure 2A can be rearranged and presented in terms of the relative turnover, whereby the enrichment at each experimental time point is made relative to the experimental plateau enrichment value(s) achieved (Figure 2B), resulting in identical *k* and *t*_1/2_ calculations, as in Figure 2A. Importantly, from a biological perspective, the near identical DNA and protein synthesis rates seen in rapidly-proliferating C2C12 myoblasts (*t*_1/2_ ~16 h) strongly suggests that protein synthesis is largely driven by cell division. This seems like a logical finding as the stimulation of cell division requires both DNA and proteome duplication; thus, these two critical processes need to be highly coordinated.

### 3.2. Comparison of Biomass Synthesis Rates under Mitotic and Post-Mitotic Conditions in C2C12 Cells

**Figure 3:**
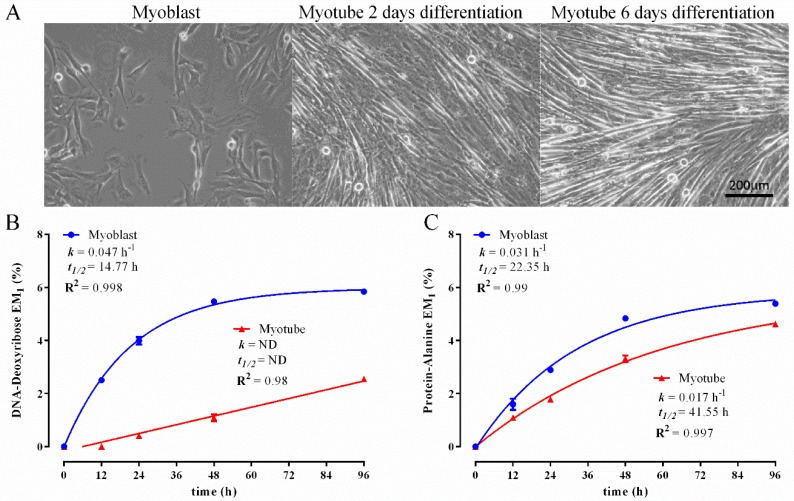
Comparison of ^2^H-incorporation into DNA-derived deoxyribose and protein-derived alanine in C2C12 myoblasts and myotubes following incubation in 4% ^2^H_2_O treatment over 96 h. Representative images of myoblast and differentiating myotubes (**A**); excess molar enrichment in the M_1_ isotopomer (EM_1_) over time in DNA-derived deoxyribose (**B**) and protein-derived alanine (**C**) in myoblasts and myotubes. Inset: fractional synthesis rate constant (*k*), half-life (*t*_1/2_) and goodness-of-fit (R^2^) from non-linear curve fitting. Two replicates were performed for each time point. Error bars represent the standard error of the mean (SEM).

**Figure 4:**
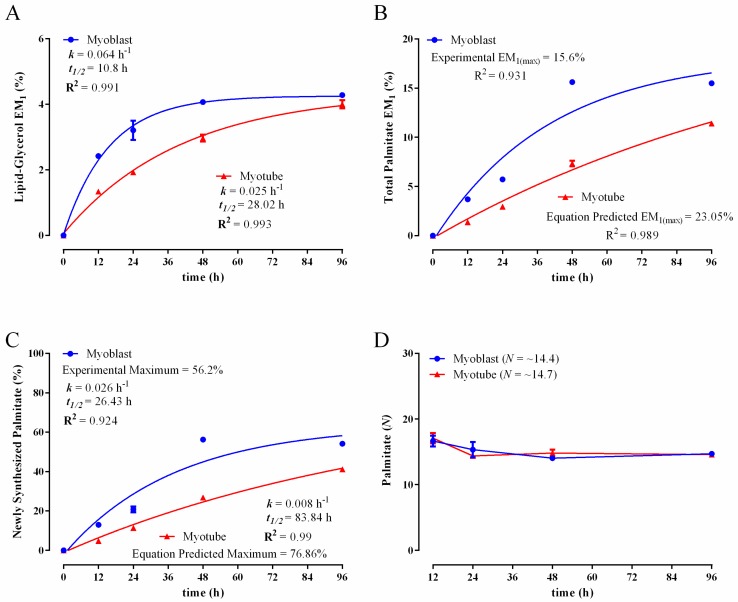
Comparison of ^2^H-incorporation into lipid-derived glycerol and total palmitate in C2C12 myoblasts and myotubes following incubation in the presence of 4% ^2^H_2_O. Excess molar enrichment in the M_1_ isotopomer (EM_1_) over time in lipid-derived glycerol (**A**) and total intracellular palmitate (**B**) in myoblasts and myotubes. Percentage of newly-synthesized palmitate in myoblasts and myotubes (**C**). The experimental maximum (plateau) value represents the percentage of the total intracellular palmitate pool that was de novo derived. For myotubes, given that plateau values were not experimentally achieved, this value was equation predicted. The maximum number of exchangeable carbon bound hydrogens (*N*) in palmitate as determined using mass isotopomer distribution analysis (MIDA) (**D**). Inset: fractional synthesis rate constant (*k*), half-life (*t*_1/2_) and goodness-of-fit (R^2^) from non-linear curve fitting. *N* values seen in the brackets of the legend (D) are an average of those obtained at the 48- and 96-h time points; these exhibited the greatest amount of labelling and, therefore, permit the most accurate calculation of *N* using MIDA. Two replicates were performed for each time point. Error bars represent the standard error of the mean (SEM).

To further validate the fractionation procedure and analytical methodology, we next determined how biomass synthetic rates differ under different growth/physiological conditions. Therefore, we compared ^2^H_2_O labelling kinetics in both C2C12 myoblasts and myotubes (Figure 3A). Unlike myoblasts, which undergo rapid proliferation and accordingly have high biomass synthetic rates, myotubes are non-dividing. As expected, near identical myoblast DNA synthesis/cell proliferation rates were measured in both experiments (Figure 3B: *k* = 0.047 h^−1^; *t*_1/2_ = 14.77 h vs. Figure 2A: *k* = 0.044 h^−1^; *t*_1/2_ = 15.69), highlighting the high degree of reproducibility between experiments (Figure 3 and Figure 4). In contrast, myotubes displayed a dramatic reduction in DNA synthetic rates, with no labelling detected in the first 12 h of ^2^H_2_O treatment (Figure 3B). This indicates that in the first 12 h of experimentation, no new cells could be detected in the myotube cultures, while in the myoblast cultures ~45% of cells were newly formed. However, when looking across the entire time course (Figure 3B), it could be clearly seen that the myotube cultures did display a small degree of DNA synthesis, indicating the presence of a minor population of cells that were actively dividing. Since the myotube DNA ^2^H enrichment pattern displayed linear kinetics and was far from reaching the plateau (Figure 3B), it was not possible to perform non-linear curve fitting to determine *k*. However, it can be seen that DNA ^2^H enrichment at the final time point (96 h) in the myotube cultures was approximately equal to that seen in the myoblasts at the 12-h time point (Figure 3B), thus indicating that on average, myoblast DNA synthesis proceeded ~8-times more rapidly than in the myotubes. It is important to note that a large body of literature has shown that when proliferating C2C12 myoblast cultures are induced to differentiate through serum withdrawal, a significant proportion of these cells escapes terminal differentiation and, thus, does not fuse to form myotubes [50,51,52]. These cells have been referred to as ‘reserve cells’, are thought to be quiescent and closely related to satellite stem cells found in skeletal muscle, which are involved in the regeneration of adult muscle in vivo [50,51,52]. Thus, the persistence of DNA synthetic activity, albeit at profoundly low rates in the myotube cultures is entirely consistent with the existence of these ‘reserve cells’; however, our data suggest that this unique cell population is not quiescent, but rather slowly dividing.

We also compared the protein synthetic activity between cultured myoblasts and myotubes. Rapidly proliferating myoblasts displayed two-fold higher rates of protein synthesis than the myotubes (Figure 3C: myoblast *k* = 0.031 h^−1^, *t*_1/2_ = 22.35 h; myotube *k* = 0.017 h^−1^; *t*_1/2_ = 41.55 h). These data show that myotubes are metabolically active and continue to actively turn over their proteome despite being largely post-mitotic.

Simultaneous measurement of ^2^H labelling in the glycerol and fatty acid moieties of total lipid extracts provided additional insight into the physiological/metabolic state of myoblasts and myotubes. ^2^H incorporation into the glycerol backbone of esterified lipids (phospholipids and glycerides) provides a measure of the rate of synthesis or turnover of the lipid pool, which largely resides in the plasma and organelle membranes of most cells. As seen in Figure 4A, the labelling kinetics of lipid-derived glycerol showed that lipid synthesis was more rapid in both myoblasts and myotubes than when compared to the other biomass components. Specifically, plateau labelling was achieved within the 96-h labelling period in both cell types (Figure 4A). Consistent with their rapid proliferation, myoblast lipid synthesis occurred almost three-fold more rapidly than in the myotube cultures (Figure 4A: myoblast *k* = 0.064 h^−1^, *t*_1/2_ = 10.8 h; myotube *k* = 0.025 h^−1^; *t*_1/2_ = 28.02 h). These data further demonstrate that non-dividing C2C12 myotubes are metabolically active and replace the equivalent of their entire membrane lipid pool every 96 h.

Additionally, analysis of ^2^H-labelling of total cellular palmitate provides an indication of de novo lipogenesis (DNL). In myoblasts, ^2^H-palmitate labelling rapidly reached a plateau after 48 h. In contrast, ^2^H-labeling in myotubes increased more slowly and had not plateaued over 96 h (Figure 4B). The calculation of DNL is complicated by the fact that most cells can also take up fatty acids from the medium (as discussed in the Methods Section), resulting in a lower maximum labelling than expected if all of the cellular fatty acid needs of the cell were generated by de novo synthesis [22,23]. This can be addressed by performing MIDA to determine experimentally the true theoretical maximum enrichment in palmitate and, therefore, to determine what portion of palmitate was newly synthesized. As has been the case in all other biomass components measured, the myoblasts synthesized palmitate more rapidly than did the myotubes (Figure 4C: myoblast *k* = 0.026 h^−1^, *t*_1/2_ = 26.43 h; myotube *k* = 0.008 h^−1^; *t*_1/2_ = 83.84 h). In the myoblasts, the experimentally-determined maximum (plateau), also referred to as the equilibrium level of labelling, was ~56% and occurred within 48 h of ^2^H_2_O treatment (Figure 4C). This indicates that ~56% of the total palmitate pool was ‘de novo synthesized’, and the rest (~44%) was derived from the culture media. Since the ^2^H enrichment kinetics did not reach equilibrium levels in the myotubes, we determined the equation predicted maximum value (Figure 4C; ~77%; R^2^ = 0.99). Thus, it appears the myotubes were more dependent on the DNL pathway than the myoblasts, with ~77% of the palmitate being ‘self-made’ and ~23% taken up from media. These differences may be hardwired into the metabolic networks of each cell type or it could reflect differences in the fatty acid content of the media. Specifically, myoblasts are grown in serum-rich medium containing 10% FBS, while myotubes are formed and maintained under serum-deprived conditions (2% horse serum). Myotubes may therefore be more reliant on the DNL pathway to supply fatty acids for vital functions, such as membrane synthesis/remodelling. Additionally, having performed MIDA analysis, we calculated the *N* values for palmitate under our experimental conditions. As can be seen in Figure 4D, myoblast and myotube *N* values did not differ and were remarkably consistent over the entire experimental time course. Given *N* was consistent over time, the data strongly suggest that the precursor pools for palmitate synthesis (H_2_O, NADPH and acetyl-CoA) were rapidly labelled and in equilibrium with ^2^H_2_O [22,27]. The *N* values shown in the legend of Figure 4D are the average of those determined at the 48- and 96-h time points, as these reflect the most accurate calculations possible due to the high degree of palmitate ^2^H enrichment at these time points. Since the calculation of *N* requires the use of consecutive isotopomer ratios (M_2_/M_1_), it is necessary that sufficient labelling (particularly in the M_2_ isotopomer) is achieved in the molecule of interest in order to attain reliable calculations [47]. The finding that palmitate *N* values in our C2C12 cells experiments ranged from 14–15 is similar to that previously reported in the literature in HepG2 and MCA cancer cells (*N* = 16–18) [23]. Interestingly, a common feature and advantage of using ^2^H_2_O to label endogenously-synthesized molecules, such as DNA, protein and lipids, lies in the amplification effect between precursor and product enrichments [16,17,24,30,31,35]. Specifically, the amount of ^2^H enrichment (EM_1_) in various molecules can exceed the ^2^H_2_O level, thus enhancing analytical accuracy. The reason for such an amplification results from the fact that several ^2^H atoms can incorporate into the analyte (i.e., multiple C–H bonds within a molecule) [16,17,24,30,31,35]. This is particularly evident in molecules with a large number of C-H bonds, such as fatty acids, as seen in the myoblast palmitate data (Figure 4B) where the plateau EM_1_ values reached ~15.6% while the ^2^H_2_O enrichment was only 4%. It is also worthy to mention that the amplification effect appears to be more pronounced in vivo in animals/humans than in the in vitro cell culture setting, likely due to the existence of additional pathways for incorporating ^2^H into product molecules in a multi-organ system [21]. This highlights the importance of performing rise to plateau experiments (at least initially) in order to establish the maximal labelling that can be achieved under the specific experimental conditions being performed.

### 3.3. Comparison of Biomass Synthesis Rates in Colon Cancer Cell Lines

**Figure 5:**
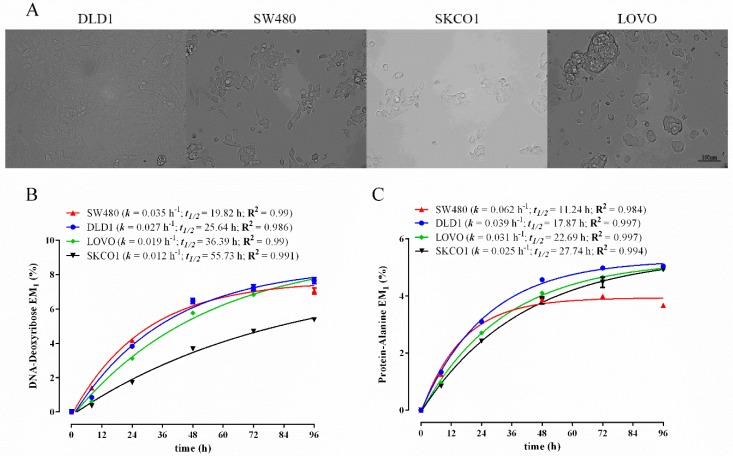
Comparison of ^2^H-incorporation into DNA-bound deoxyribose and protein-derived alanine in colon cancer cell lines following incubation in the presence of 5% ^2^H_2_O over 96 h. Representative images of colon cancer cell lines (**A**). Excess molar enrichment in the M_1_ isotopomer (EM_1_) over time in DNA-derived deoxyribose (**B**) and protein-derived alanine (**C**) in human colon cancer cell lines grown under identical conditions. Inset: fractional synthesis rate constant (*k*), half-life (*t*_1/2_) and goodness-of-fit (R^2^) from non-linear curve fitting. Three replicates were performed for each time point. Error bars represent the standard error of the mean (SEM).

Determining the rates at which cells grow and turnover is fundamentally important in cancer research. Therefore, we wanted to validate our biomass fractionation method to characterize the growth and turnover characteristics of a panel of human cancer cell lines using ^2^H_2_O labelling. We selected a panel of adherent human colon cancer cell lines; DLD1, SW480, SKCO1 and LOVO [53]. In order to compare the biomass synthesis kinetics of these cells directly to one another, all of the cell lines were cultured using the same culture media conditions (DMEM in 10% FBS). The ^2^H labelling of DNA-derived deoxyribose revealed substantial differences in DNA synthesis rates (*k*) and calculated cell doubling times (*t*_1/2_) between the various cell lines (Figure 5B). In particular, SW480 cells were found to proliferate almost three-times faster than SKCO1, with the other cells lines having intermediate replication rates. To validate the cell doubling times obtained using ^2^H_2_O labelling and non-linear curve fitting (*t*_1/2_), we additionally performed manual hemocytometer-based cell counting experiments with the DLD1 and SW480 cell lines grown in the same culture media, but in the absence of ^2^H_2_O. Importantly, manual cell counting yielded near identical cell doubling times to that obtained using *t*_1/2_ values (DLD1: *t*_1/2_ ~25.6 h, manual ~24 h; SW480: *t*_1/2_ ~19.8 h, manual ~20 h), indicating that the ^2^H_2_O method was accurate and did not have any growth-impeding effects. Interestingly, we observed a high correlation between the rate of protein synthesis and DNA synthesis across this panel of cancer cells (Figure 5B,C). For example, similarly to DNA synthesis, SW480 had ~2.5-fold higher protein synthetic rates than SKCO1. It is worth noting, however, unlike that seen in the proliferating C2C12 myoblasts, where rates of DNA and protein were similar (Figure 2 and Figure 3), all of the cancer cell lines displayed higher rates of protein turnover than DNA replication (i.e., lower *t*_1/2_ values in protein compared to that in DNA). This was most evident in the SKCO1 cell line (Figure 5A,B: DNA *t*_1/2_ = 55.7 h; protein *t*_1/2_ = 27.7 h; two-fold difference). This indicates that cancer cells may exhibit a high degree of protein remodelling consistent with enhanced autophagy and proteasome activity [54]. Lipid biosynthesis, as revealed by ^2^H labelling of lipid-derived glycerol, also reflected overall rates of cell division, but occurred at a higher rate than DNA replication in these cancer cells (average *t*_1/2_ for DNA across all four lines = 34.4 h; average *t*_1/2_ for lipid-glycerol = 11.6 h). Thus, it can be estimated that on average, during the course of one cell division event, the esterified (stored) lipid pool turned over three times, indicating a large degree of membrane remodelling. Such rapid lipid turnover was not evident in the non-malignant C2C12 myoblast cell line (Figure 3A and Figure 4A: DNA *t*_1/2_ = 14.8 h; lipid-glycerol *t*_1/2_ = 10.8 h).

**Figure 6:**
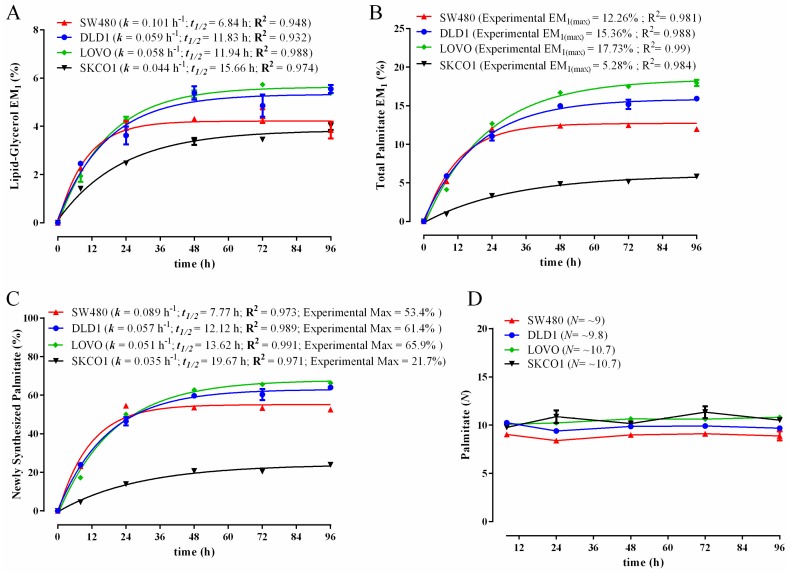
Comparison of ^2^H-incorporation into lipid-derived glycerol and total palmitate in colon cancer cell lines following incubation in 5% ^2^H_2_O for 96 h. Excess molar enrichment in the M_1_ isotopomer (EM_1_) over time in lipid-derived glycerol (**A**) and total intracellular palmitate (**B**) in human colon cancer cell lines grown under identical conditions. Percentage of newly-synthesized palmitate in colon cancer cell lines (**C**). The experimental maximum (plateau) value represents the percentage of the total intracellular palmitate pool that was de novo derived. The maximum number of exchangeable carbon bound hydrogens (*N*) in palmitate as determined using MIDA (**D**). Inset: fractional synthesis rate constant (*k*), half-life (*t*_1/2_) and goodness-of-fit (R^2^) from non-linear curve fitting. *N* values seen in the brackets of the legend (D) are an average of those obtained at the 48-, 72- and 96-h time points; these exhibited the greatest amount of labelling and, therefore, permit the most accurate calculation of *N* using MIDA. Three replicates were performed for each time point. Error bars represent the standard error of the mean (SEM).

As with the C2C12 analysis, we also determined ^2^H labelling levels and kinetics in the total intracellular palmitate pool in the colon cancer cell lines. As depicted in Figure 6B,C, ^2^H enrichment was rapidly achieved, with plateau (steady) levels occurring within 48 h in all four cell lines. Consistent with the other biomass components, the most rapidly dividing colon cancer cells displayed the most rapid palmitate labelling (Figure 6C). Interestingly, there was a pronounced difference in the reliance on the DNL pathway between the SKCO1 cells and that of the other lines. Specifically, the SKCO1 cells displayed markedly lower ^2^H enrichment than the other lines, with ~22% of the cellular palmitate pool being DNL derived (Figure 6C), whereas DNL contributions were markedly higher in the other cell lines (Figure 6C: DLD1 ~61%; SW480 ~53%; LOVO ~66%). Furthermore, as with the C2C12 myoblasts and myotubes, upon performing MIDA analysis of palmitate enrichment, *N* values were highly stable in each colon cancer cell line over time, indicating rapid and consistent precursor labelling (Figure 6D). However, the measured palmitate *N* values in all four colon cancer cell lines (~10 on average) were substantially lower than that which we had measured in the non-malignant C2C12 cells (~14.5) or to that found by others in HepG2 and MCA cancer cells (*N* = 16–18) [23]. This suggests that the colon cancer lines display a reduction in hydrogen atom exchange rates between cellular H_2_O and the NADPH and/or acetyl-CoA precursor pools. The reason for this remains unclear. It is worthy to mention that our findings of varied reliance on the DNL pathway between different colon cancer cell lines in vitro do not support the commonly accepted notion in the literature that cancer cells (and proliferating cells in general) are predominantly dependent on the de novo synthesis of fatty acids [55,56,57]. It is clear from our data that in the colon cancer cell lines and also in the rapidly proliferating C2C12 myoblasts that quantitatively significant amounts of the stored (esterified) fatty acid pool was serum and not de novo derived. In fact, in the case of the SKCO1 cancer cell line, the vast majority (~78%) of intracellular palmitate was exogenously derived. This capacity to use exogenous extracellular fatty acids for complex lipid synthesis has also been recently demonstrated by others in a range of rapidly dividing cell lines in vitro [58]. Conversely, in our predominantly non-dividing C2C12 myotube cultures, the reliance on the DNL pathway was relatively high (~77% palmitate was ‘self-made’), thus indicating the reliance on the DNL pathway is unlikely related to cell proliferation. The ^2^H_2_O labelling technique and extraction method described here could easily be applied to 3D culture systems and is also ideally suited for studying in vivo tumor growth and metabolism in pre-clinical animal models [59,60] and human clinical settings [36,61].

## 4. Conclusions

Taken together, we present here a cell fractionation/extraction method to combine with ^2^H_2_O labelling and GC-MS analysis in order to simultaneously measure different cellular biomass synthesis rates from the one sample. Using this method, we were able to validate that in actively-proliferating mammalian cells, biomass synthesis rates are strongly linked to the rate of cell division, thus demonstrating the utility of the approach. Furthermore, in both proliferating and non-proliferating cells, compared to DNA and protein, it is the lipid pools that undergo the most rapid turnover. Finally, our data reveal a marked heterogeneity in the reliance on the DNL pathway in a panel of different human colon cancer cell lines.

## Supplementary Materials

The following are available online at http://www.mdpi.com/2218-1989/6/4/34/s1.
