# Supplementary material for: Analysis of Mammalian Cell Proliferation and Macromolecule Synthesis Using Deuterated Water and Gas Chromatography-Mass Spectrometry
Source: Metabolites. 2016 Oct 13;6(4):34. doi: 10.3390/metabo6040034 (PMC5192440; doi:10.3390/metabo6040034)
Supplement: Supplementary file 1 [file metabolites-06-00034-s001.pdf]

Supplementary Materials: Analysis of Mammalian Cell Proliferation and Macromolecule Synthesis Using Deuterated Water and Gas Chromatography-Mass Spectrometry

Victoria C. Foletta, Michelle Palmieri, Joachim Kloehn, Shaun Mason, Stephen F. Previs, Malcolm J. McConville, Oliver M. Sieber, Clinton R. Bruce and Greg M. Kowalski

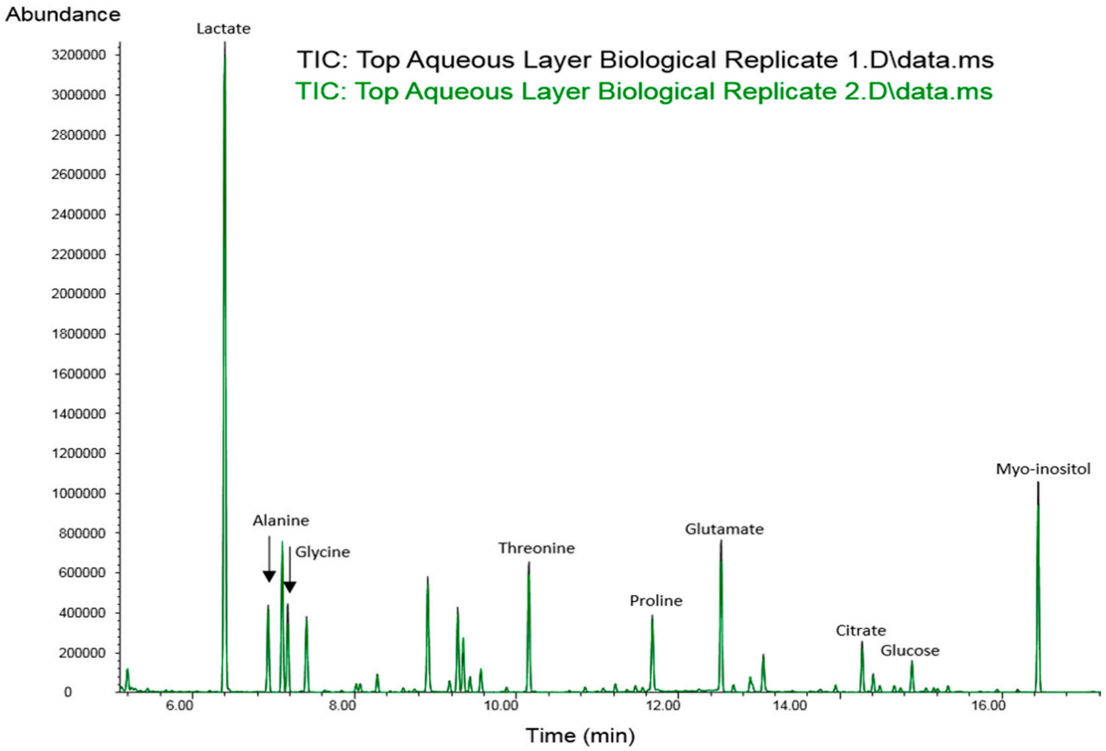

Figure S1. Total ion chromatogram (TIC) of the top aqueous layer generated using the described biphasic extraction procedure. Two biological replicates from C2C12 myoblasts were analysed via GC-MS using the MOX-TMS derivatizing strategy, confirming the presence of polar intracellular metabolites.
